# Pneumonia among under – five children in Ethiopia: a retrospective analysis from an urban hospital

**DOI:** 10.21203/rs.3.rs-2790057/v1

**Published:** 2023-04-12

**Authors:** Walelegn Worku Yallew, Selamawit Assefa, Berhane Yemane

**Affiliations:** Addis Continental Institute of Public Health; Department of Pediatrics, Yekatit 12 Hospital Medical College; Addis Continental Institute of Public Health

**Keywords:** Pneumonia, under-five children, hospital admission, mortality, Ethiopia

## Abstract

**Background:**

Pneumonia is the leading cause of death in under-five children in low-income countries. However, the burden of pneumonia in hospital admission is not traced systematically. This study was conducted to determine the proportion of under-five pneumonia admissions among children admitted to a hospital in Addis Ababa, Ethiopia between 2017–2021.

**Methods:**

A retrospective record of pediatric admissions to the Yekatit 12 referral hospital in Addis Ababa, Ethiopia was assessed for the period 2017– 2021. The date of admission and discharge, length of stay, and outcome at discharge were collected in accordance with the Ethiopian National Classification of Diseases (NCoD). Descriptive statistics were used to assess the proportion of under-five children with pneumonia. Survival analyses using Log rank test and cox regression analysis were done to assess time to recovery (recovering from illness). Multivariable logistic regression was used to assess the influence of selected factors on pneumonia associated hospital admission.

**Results:**

Between 2017–2021, 2170 children age 1 to 59 months were admitted, 564 (25.99%; 95% confidence interval 24.18% to 27.87%) were diagnosed with pneumonia. Among the sixty children who died during their hospitalization, 15 had been diagnosed with pneumonia. The median time to recover from pneumonia and discharge was 6 days. The odds of pneumonia hospital admission were higher among younger children (4.36 times higher compared to elder children with 95% CI 2.77,6.87)and were increased between the months of September to November.

**Conclusions:**

Pneumonia accounts for more than a quarter of hospital admissions in under-five children and for a quarter of deaths in this urban cohort. Hospital admission due to pneumonia was higher among older children (36–59 months of age) in the months following the heavy rain months (September to November) as compared to younger children. Our data strongly support increase of vaccination to prevent under 5 pneumonia.

## Background

Pneumonia is the leading cause of death among children under five years of age in Sub-Saharan Africa [1] as compared to other infectious diseases [2]. In Ethiopia, the prevalence of pneumonia in under-five children is high[3–5]. Evidence indicate that community-acquired under-five children’s pneumonia is 20.68%[6]. Studies have also shown that pneumonia accounts for 38.6% of the total emergency ward admissions [7] and is the leading cause of mortality in referral hospitals[7]. The common causes of pneumonia in children are viruses accounting for 61.4%, followed by bacteria (27.3%) [8].

The introduction of pneumococcal conjugate vaccines significantly reduced childhood pneumonia hospitalization[9, 10]. Ethiopia introduced the Hemophilus influenzae type b (Hib) and pneumococcal conjugate vaccine (PCV 13) into the nation’s infant immunization programme in 2007 and November 2011, respectively[11]. However, according to the 2019 Ethiopian Demographic and Health Survey (EDHS), only 44% of children nationally and 83.3% in Addis Ababa received all basic vaccination[12]. Several risk factors are identified for under-five children community-acquired pneumonia, including seasonal variation[13–16], vaccination status, nutritional status[17] and children living in crowded households with economic stress [18]. In turn, increased length of hospital stays for admitted children may increase the financial burden to the family[19], and is a particular issue in a developing country such as Ethiopia. In India for example almost half of household’s total expense is due to hospital stay for a single episode of severe pneumonia[20].

The epidemiology of pneumonia can significantly vary by many factors, including the availability of preventive health services and climatic conditions[15]. Hence, contextual data are critical to implementing relevant interventions. Therefore, this study was conducted to examine the under five children pneumonia-related admission and identify factors that influence admission in Addis Ababa.

## Methods

### Design and setting

Admissions to the department of Pediatrics in Yekatit 12 Hospital for the period 2017–2021 were retrospectively reviewed. Yekatit 12 Hospital has a total of 350 beds, of which 75 are allocated for the pediatric and 50 for neonatal wards. The hospital provides outpatient and inpatient health care services and serves as a referral center to burn injury [21, 22].

### Participants

All under-five children admitted to the pediatric ward between 2017–2021 were included in the analysis (N = 2170). Children whose treatment outcome was unknown were excluded from the analysis. Preterm and neonates were also excluded from the analysis.

A data abstraction form was used to extract information from hospital records. The information gathered includes the child’s age and sex, address, date of admission and discharge, length of stay, and condition at discharge or death. The information was collected in accordance with the Ethiopia National Classification of Diseases (NCoD). Data were collected by trained data collectors using the Open Data Kit (ODK) mobile application. The data were collected after ethical approval from Addis Continental Institute of Public Health Institutional Review Board (IRB) and Yekatit 12 Hospital Medical College.

### Data Management and Statistical analysis

Data were extracted from ODK and exported to STATA for analysis. The proportion of pneumonia under five children admission was calculated as the number of children admitted with pneumonia divided by all under-five children admitted to the hospital in the same study year. Descriptive statistics were used to assess the proportion of under-five children with pneumonia.

Time to recovery was computed with median with Interquartile range. The Log rank test and Proportional Hazards (PH) assessment were computed to compare the survival curves of pneumonia and without pneumonia children to time to treatment recovery. The Wilcoxon-Gehan-Breslow (WGB) test was used to determine whether there was a relationship between sex, age, and admission month to time to recovery from admission cases. In addition, time to recovery and relationship between pneumonia was assessed using cox regression analysis.

Multivariable logistic regression was used to examine the relationship between Under-five children’s pneumonia admission and age of the child, gender, and season. A P-value less than 0.05 was considered statistically significant. Missing data were treated using the multiple imputation method assuming data were missing at random.

## Results

### Socio-Demographic Characteristics

A total of 2170 children were admitted to the hospital from January 2017 to December 2021 and included in the analysis. The mean age of children was 20 months with a standard deviation of + 15 Months (Table 1).

### Under five children Pneumonia Admission status and time to Recovery

From the total admissions from January 2017 and December 31, 2021 (n = 2170), 564 under-five children with pneumonia (25.99%, with 95% confidence interval of 24.18–27.87%) were admitted. Out of 564 pneumonia cases, 530 (93.97% were severe community-acquired pneumonia and the remaining 34(6.03%) were mild pneumonia cases.

The mean length of stay in the ward for all admitted children was 9 days + 14 days standard deviation (SD). This was used as a measure of mean time to recovery. Sixty children (2.76%) children died and among this group, with pneumonia the cause of death for 15 (25%).

There was a difference between children admitted with pneumonia and children admitted without pneumonia in their time to discharge, 5 days for children diagnosed with pneumonia vs, 10 days for children who did not have a diagnosis of pneumonia (p < 0.001). The time to recover/discharge after admission without pneumonia was therefore increased by 1.56 times as compared to children admitted with pneumonia (95% CI: 1.36, 1.79) (Table 2).

### Factors associated with Under five children Pneumonia admission

The association between pneumonia admissions and gender, age in months, and months of admission in the hospital in under-five children was assessed using multivariate analyses. The gender of the child was not statistically significant with pneumonia admission status. Under five children’s pneumonia admissions were significantly associated with age at the time of admission. Children 1–11 months of age were 4.36 times more likely to be admitted compared to children 48–59 months of age. Overall, the odds of pneumonia admission decline as the child’s age increased. The odds of admission due to pneumonia were greater in September, October, and November months. Furthermore, children admitted in September were 1.96 times more likely to be diagnosed with pneumonia compared to children admitted in January; AOR 1.96; 95% CI 1.22,3.15 (Table 3).

## Discussion

A quarter of children under five years of age who were admitted to the pediatric ward of the Yekatit 12 Hospital in Addis Ababa between 2017 and 2021 were diagnosed with pneumonia. The time to recover was significantly longer in pneumonia cases compared to non-pneumonia cases admitted in the hospital. The odds of being admitted to the hospital due to pneumonia was significantly greater among younger children and among those admitted during the months between September and December.

The proportion of admissions due to pneumonia in this study was higher than a similar retrospective study (18.5%) conducted among under five children admitted to the University of Gondar Referral Hospital[23]. However, our observations were similar to a study conducted at Zewditu Memorial Hospital, in Addis Ababa, which found pneumonia to account for 25.3% of admissions [7]. Furthermore, to a study from Black Lion (Tikur Anbessa) Hospital Pediatric Emergency Department reported 26.7% mortality due to pneumonia in under five children [24]. Here, we similarly found that a quarter of deaths that occurred in children admitted to Yekatit 12 Hospital occurred in children diagnosed with pneumonia. Taken together, the findings indicate pneumonia continues to be a major cause of hospital admission for under-five children despite the introduction of Hemophilus influenzae type b (Hib) and pneumococcal conjugate vaccine (PCV 13) vaccines into the nation’s infant immunization programme [11], and is a major cause of death. We note that we did not have access to vaccination records of this cohort.

In this study we found that children under five children with pneumonia took approximately 6 days to recover. This finding is higher than other studies conducted in Ethiopia [25, 26], where hospital stay of more than 5 days is considered to be high [19]. Previous studies have found that prolonged hospital stay are strongly associated with socio-demographic factors including, the presence of co‐morbidity and malnourishment in patients[25–27]. Notably, prolonged stay in the hospital creates a high burden for the family and to the health institution[28], and is associated with increased risk of developing drug adverse reaction and hospital acquired infection[29].

We observed younger children were more likely to be admitted for pneumonia than older children, which is consistent with in previous studies[30, 31]. This may be younger children’s immune systems are immature and increase vulnerability to respiratory tract infections[32, 33]. Furthermore, pediatric PCVs have a possible indirect impact of preventing pneumococcal pneumonia in older children[34]. Despite the evidence indicating that hospital admissions for severe acute lower respiratory infections are higher in boys than in girls [7], we did not observe any sex difference in our study. This may be due to very low gender difference in child health service utilization in Ethiopia[35].

Variability in humidity and temperature [36], and etiologic factors like bacterial *Mycoplasma pneumoniae* [37] are attributed to the increase in pneumonia cases in certain seasons. Studies indicate that weather conditions increase the risk of pneumonia[38]. Furthermore, pneumonia-related admission and mortality among under-fives are higher in the rainy season [16]. In contrast, some evidence has shown that the risk of pneumonia was higher in the dry season than in the rainy season[15]. In this study, pneumonia admission was high from September to November, which is a semi-rainy season for the study area.

Limitations of this study include it being a retrospective record review, with some important factors not fully documented (missing records) nor available, including vaccination status and etiology of the pneumonia. Thus, information biases and residual confounding could be factors in our conclusions. In addition, the study may not be generalizable to all under five children, so it needs wider and prospective longitudinal study is necessary to obtain more complete information.

## Conclusions

In conclusion, pneumonia accounted for more than a quarter of admissions and deaths in this cohort of under five children evaluated in an urban hospital in Addis Ababa. Time to recovery was higher than previously reported in Ethiopia and the odds of pneumonia admissions were higher in the months from September to November, and among younger children. Preventive measures such as vaccination should be taken before pneumonia season begins to prevent hospitalizations and poor outcomes.

## Figures and Tables

**Table 1:** Sociodemographic Characteristics for children under five from 2017 to 2021. (January 2017 to December 2021)

Characteristics		Number	Percent
**Sex**	Male	1264	58.25
	Female	906	41.75
**Age in Months**	Age 1–11 Months	752	34.65
	Age 12–23 Months	506	23.32
	Age 24–35 Months	436	20.09
	Age 36–47 Months	248	11.43
	Age 48–59 Months	228	10.51
**Month of admission**	January	154	7.10
	February	132	6.08
	March	135	6.22
	April	76	3.50
	May	93	4.29
	June	79	3.64
	July	119	5.48
	August	151	6.96
	September	291	13.41
	October	398	18.34
	November	322	14.84
	December	220	10.14

**Table 2:** Time to recover from Admission to under five children at Yekatit 12 teaching Hospital from 2017 to 2021 in Addis Ababa, Ethiopia.

Characteristic	Recovery		Crude HR (95% CI)	Adjusted HR^2^ (95% CI)
	Event	Censored^1^		
**Admission type**				
Without Pneumonia	1006	600	1.54 (1.35, 1.75)***	1.56 (1.36,1.79)***
Pneumonia	306	256	1	1

NB:

1 Children who died, same, left, transferred, absconded

2 Model adjusted for sex, Age of children, Month of admission.

**Table 3:** Gender, age, and time of admission in relation to under-five children pneumonia admission from 2017 to 2021 in Addis Ababa ,2019, Ethiopia

Characteristic	Under five children Pneumonia admission		Crude OR (95% CI)	Adjusted OR^1^ (95% CI)
	Yes	No		
**Gender**					
Male	331	933	1	1
Female	233	673	0.98 (0.80,1.19)	0.98 (0.79,1.20)
**Age in Month**					
Age 1–11 Months	254	498	4.33 (2.77,6.79)***	4.36 (2.77,6.87)***
Age 12–23 Months	160	346	3.93 (2.47,6.24)***	4.09 (2.56,6.53)***
Age 24–35 Months	97	339	2.43 (1.51,3.93)***	2.42 (1.49,3.93)***
Age 36–47 Months	29	219	1.13 (0.63,1.99)	1.20 (0.67,2.15)
Age 48–59 Months	24	204	1	1
**Month admissions**					
January	31	123	1	1
February	26	106	0.97 (0.54,1.74)	1.02 (0.56,1.85)
March	25	110	0.90 (0.50,1.62)	0.89 (0.49,1.61)
April	2	74	0.11 (0.02,0.46)***	0.12 (0.03,0.51)***
May	13	80	0.64 (0.32,1.31)	0.72 (0.35,1.46)
June	9	70	0.51(0.23,1.13)	0.53 (0.24,1.18)
July	20	99	0.80 (0.43,1.49)	0.85 (0.45,1.60)
August	38	113	1.33 (0.78,2.29)	1.37 (0.79,2.37)
September	95	196	1.92 (1.21,3.06)***	1.96 (1.22,3.15)***
October	131	267	1.95 (1.24,3.04)***	2.06 (1.32,3.25)***
Gender				
November	116	206	2.23 (1.42,3.52)***	2.33 (1.47,3.71)***
December	58	162	1.42 (0.86,2.33)	1.42 (0.86,2.35)

NB:

1 Model adjusted for under five children pneumonia admission; sex, season.

*** p < .001,

** p < .01,

* p < .0.5

## Data Availability

The datasets used and/or analyzed during the current study are available from the corresponding author on reasonable request.
